# Translation

**Published:** 1881-04

**Authors:** 


### TRANSLATION.

EXPERIMENTAL RESEARCHES UPON CONGENITAL ATROPHY AND DIGESTIVE TURGESCENCE OF THE SPLEEN: BY M. MASSOIN.—*Bulletin de L'Acad, Royale De Medicine De Belgique*. No. 11. Vol. XIV., 3rd Series. BRUSSELLS, 1880.*

[Translated by F. Peyre Porcher, M. D., Charleston, S. C., for the INDEPENDENT PRACTITIONER.]

After experiments made upon several generations of rabbits by removing the spleen, which are detailed in full in his paper, the author gives the following conclusions:

1. Rabbits from which the spleen has been removed artificially produce offspring which possess a spleen, but this organ is atrophied.

2. The splenic atrophy is not constant in the offspring of couples, the spleens of which have been removed.

3. In the second generation this atrophy is not more marked than in the first.

4. A moderate repast does not seem to cause turgescence of the spleen.

5. After a very large meal which is given to a very hungry animal, the spleen attains its maximum of turgescence about five hours after the meal.

6. We have never found that the spleen was reproduced when the extirpation had been complete in rabbits and dogs, which confirms the observations of Peyrani, but is contrary to the earlier assertions of Philipean.

7. We have never, so far, met the ganglionic hypertrophy which, it has been asserted, is established after splenotomy, to realize a supplementary function. In 1871 we had observed the left axillary "ganglions" acquire and preserve a considerable size in a dog deprived of its spleen; but this result seemed to be owing to a local irritation which existed for sometime in the abdominal wound; it was an inflammatory engorgement and nothing else.

In our first paper we sustained, notwithstanding the doubt of Forster, the possibility of finding congenital absence of the spleen in a human subject, otherwise perfect. Since then a case has been recorded which no longer leaves the question in doubt; it is that observed by M. M. Koch and Wachsmuth at Altona.†

*Recherche experiementales sur l'atrophic congenitale et la turgescence digestive; de la Rate par M. Massoin, membre titulaire.

†Berlin Klin. Wochenschrift, 10 fevrier, 1879.

